# The serologic investigation and viral isolation of bluetongue virus in Shangri‐La in Southwest China

**DOI:** 10.1111/tbed.13292

**Published:** 2019-07-22

**Authors:** Ying Liang Duan, Hai Sheng Miao, De Fang Liao, Mei Ling Kou, Zhan Hong Li, Zheng Wang, Hua Chun Li, Le Li

**Affiliations:** ^1^ Yunnan Tropical and Subtropical Animal Virus Diseases Laboratory Yunnan Animal Science and Veterinary Institute Kunming Yunnan China; ^2^ Zhong Dian Animal Disease Control Center Shangri‐La Yunnan China

**Keywords:** bluetongue virus, China, serotype 21, Shangri‐La, yak

## Abstract

Bluetongue is an arthropod‐borne viral disease of ruminants caused by bluetongue virus (BTV). In China, BTV is relatively common in Yunnan Province with the exception of northern regions around Shangri‐La, where the average altitude is approximately 3,450 metres. Recently, the seroprevalence of BTV has been measured in yaks in Shangri‐La; therefore, this study investigated BTV infections in this area. The serological investigation in five villages in Shangri‐La showed that there were sporadic BTV infections in yaks (20 of 507 positive) during 2014 to 2017, while the seroprevalence of BTV at three goat farms in a nearby river valley was 35%–65% in 2017. Subsequently, 20 sentinel goats were kept on two separate farms in the river valley and monitored for seroconversion between May and September of 2017. Five of the sentinel animals were tested positive for antibodies to BTV by C‐ELISA during the study period, and 13 BTV isolates were isolated from ten sentinel animals. All isolates were identified as the same serotype, and the complete nucleotide sequence of one was determined. The genomic sequences showed that the isolated BTV strain belonged to serotype 21 and had approximately 99.8%–100% homology with three Indonesian BTV‐21 strains (D151, RIVS‐66 and RIVS‐60) between their coding sequences (CDSs) except for Seg4 (99.5%). Besides, our data suggested that this BTV‐21 strain might have also infected some local yaks and sheep.

## 1 INTRODUCTION

Bluetongue virus (BTV) is the prototype of the genus *Orbivirus* within the family *Reoviridae*. It is the causative agent of bluetongue disease, which is an infectious, non‐contiguous and arthropod‐borne viral disease of ruminants (Roy, 2007; Shafiq, Minakshi, Bhateja, Ranjan, & Prasad, 2013). BTV was identified for the first time in South Africa in 1906 and is epidemic in tropical, subtropical and temperate areas between the latitudes of approximately 40° north and 35° south (Maclachlan, 2011). Some species of *Culicoides* are the insect vectors for BTV transmission (Gibbs & Greiner, 1994).

The BTV genome is composed of 10 segmented dsRNAs, which are named Seg1 to Seg10. The genomic RNAs encode seven capsid proteins (VP1‐VP7) and five non‐structural proteins (NS1‐NS4 and NS3a). The virion contains the 10 segmented dsRNAs and few non‐structural proteins in the core and is coated by two layers of capsids (Roy, 2007). VP2 and VP5 together construct the outer layer of the capsid, and VP2 is the critical protein for virus attachment to target cells (Roy, 2007). Accordingly, the VP2 protein has the most sequence diversity among BTV proteins and is the basis of serotype classification. To date, 27 serotypes have been identified worldwide (Jenckel et al., 2015; Maan et al., 2011), and 14 serotypes have been confirmed in China since 1979 (Kirkland et al., 2002; Yang et al., 2017).

Yunnan Province is located in a tropical and subtropical region and has diverse climates. It is the main region of BTV prevalence in China, and up to 13 serotypes of BTV have been identified in Yunnan (Kirkland et al., 2002; Yang et al., 2017). However, Shangri‐La County, which is located in northern Yunnan Province, has always been excluded from the BTV survey, because it has relatively cold weather and is closed to traffic. To our knowledge, this study is the first to investigate BTV prevalence in this region.

Shangri‐La is adjacent to the south edge of the Qinghai‐Tibetan Plateau and has an average altitude of 3,450 m. The landform is rugged because there are several rivers that flow across the region. The average annual temperature on the Shangri‐La plateau is approximately 5°C, but the climate is relatively warm in summer in the valleys where the lowest altitude is 1,900 m. Yaks, sheep and goats are the main animals raised on the Shangri‐La plateau, and yaks are the most prevalent animals raised on the highland.

Recently, anti‐BTV IgG seroprevalence in yaks and sheep was measured on the Tibetan Plateau in western China (Ma et al., 2017), and a serological investigation also discovered anti‐BTV IgG ‐positive yaks in Shangri‐La (Xiao et al., 2011). Therefore, this study was carried out to investigate the BTV seroprevalence and identify the BTV strains in Shangri‐La.

## 2 MATERIALS AND METHODS

### 2.1 Sample collection

All the animals in this study were summarized in Table S1, and the operations for sample collection were described as follows.

For the antiserum investigation, serum samples were collected from yaks by the local Animal Disease Control Center (ADCC) during 2014 to 2017 in 5 villages, and serum samples were collected from goats in 2017 from three different farms in the village of Nixi, which is near the other villages.

After that, 20 local goats less than 1 year old that were identified as BTV negative by C‐ELISA were selected as sentinel animals and assigned to two surveillance sites in Nixi. The animals were kept among other goats between May and September of 2017. Serum samples and EDTA^+^ blood from all sentinel animals were collected every 10 days and used for tests and virus isolation.

In addition, for viral nucleic acid research, EDTA^+^ blood was collected from yaks and sheep in the villages of Xiaozhongdian and Nixi in 2017, and a series of BTV strains isolated in China previously and recently were also used for BTV Seg1 fragments alignment.

### 2.2 Serologic and molecular techniques

#### 2.2.1 C‐ELISA

The C‐ELISA method was used to test the anti‐VP7 (BTV) antibodies of animal serum samples, as described previously (Chapter 2.1.3, OIE Terrestrial Manual 2009). Briefly, the lysates of BTV‐infected BHK‐21 cells were used to coat 96‐well microplates, and serogroup‐reactive anti‐VP7 mAb (8A3B6) (Kirkland et al., 2002) was used as a competing antibody. Colour was developed by TMB substrate and stopped by 1 M sulphuric acid. Optical density (OD) values were read by a microplate reader (Multiskan Ascent, Thermo). Per cent inhibition (PI) was calculated as PI = 1−OD_sample_/OD_negative_. Samples with PI ≥ 50% were considered positive.

#### 2.2.2 Serum neutralization test (SNT)

The SNT is a semiquantitative method to detect anti‐BTV specific antibodies in serum. Briefly, starting from a 1/4 dilution, serum samples were diluted in a twofold dilution series. Every diluted sample was added into 4 replicate wells at 50 μl per well. Approximately 100 TCID_50_ (50% tissue culture infective dose) of the newly isolated virus was added at a volume of 50 μl per well and mixed with the diluted serum samples. After that, 1 × 10^4^ BHK‐21 cells were added to each well in a volume of 100 μl, and after incubation for 4–6 days, the CPE was observed under an inverted microscope. The wells were scored for the degree of CPE observed. Titres were expressed as the final dilution of serum present in the serum/virus mixture when 50% of the duplicate wells were protected according to Reed–Muench methods (Reed & Muench, 1938).

#### 2.2.3 RT‐PCR

**Figure 1:**
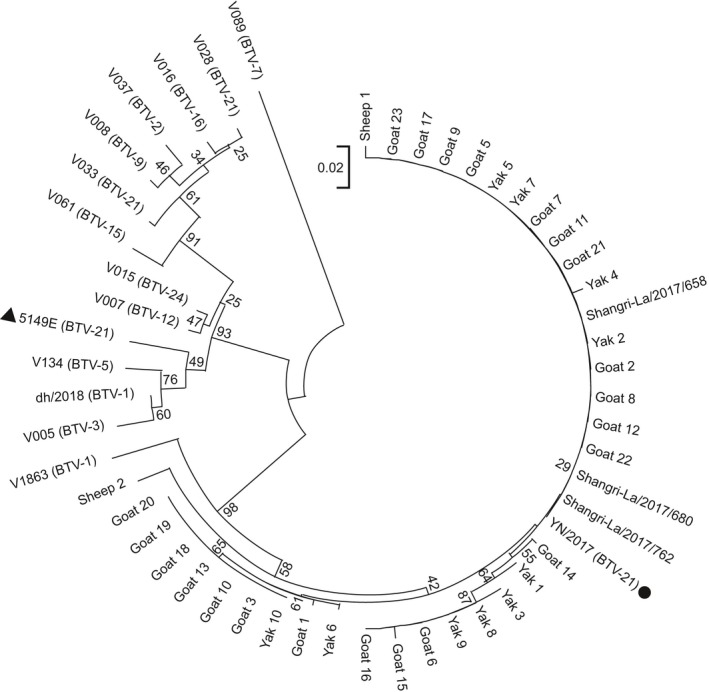
Phylogenetic analysis of the relationship among the BTV‐21 isolates and domestic BTV strains by Seg1 fragment sequence similarity. Sequence alignment was performed by *MEGA‐X*, and the evolutionary tree was built by the neighbour‐joining method (bootstrap = 1,000). (1) Shangri‐La/2017 (658, 680 and 762) and YN/2017 were the representative isolates from Shangri‐La. (2) V005, V007, V008, V015, V016, V028, V033, V037, V061, V134 and dh/2018 were the domestic strains isolated during 2012–2018, and V1863 was isolated in 1979. (3) Yak, sheep and goat represented the BTV nucleic acid amplified from these animals in Shangri‐La

For viral nucleic acid detection in animal blood, as well as in the samples of BTV strains isolated previously (see Table S2) and used for Seg1 analysis (Figure 1), high‐throughput BTV RNA extraction from the EDTA^+^ blood samples was performed by the MagMAX^TM^ Express‐96 (Ambion, USA) with a MagMAX‐96 Viral RNA Isolation Kit (Ambion, USA). A pair of universal primers, Bs1p3f (5‐TCATCGAATT TCGGGCTAA) and Bs1p3r (5‐CCCACATCTT TACAAACCAC), were used to amplify a 429 bp fragment of BTV Seg1. RT‐PCR for Seg1 was performed by a GeneAmp 9700 PCR system (Thermo) and a PS One‐Step RT‐PCR Kit ver.2 (Takara) using primers Bs1p3f and Bs1p3r according to the manufacturer's instructions.

For nucleic acid analysis of the BTV isolates from Shangri‐La, whole RNAs were extracted from BHK‐21 infected with the isolates and reverse transcribed to cDNA by SuperScript III (Thermo) with random primers. Seg2 fragments of 999 bp were amplified by Pfu (Tiangen) with primers S2‐*F* (5‐TAACGCAAAC GGAAATCGCT CA) and S2‐R (5‐GCGTTATCGG AAGATACCAA ACCA).

#### 2.2.4 Sanger sequencing and analysis

The PCR products of Seg1 and Seg2 were sent to the Kunming branch of the Tsingke Biological Technology Company for sequencing using primers Bs1p3f/Bs1p3r and S2‐F/S2‐R, respectively. Sequences in the middle of Seg1 (524–707 bp) and Seg2 (1654–2520 bp) were used for alignment.

### 2.3 Virus isolation

The virus was isolated by the egg‐C6/36‐BHK‐21 (ECB) virus isolation system, in which the serum was inoculated in an embryonated egg, *Aedes Albopictus* cell line C6/36 and baby hamster kidney cell line BHK‐21, in that order. The ECB virus isolation was performed as previously described (Clavijo, Heckert, Dulac, & Afshar, 2000). Briefly, the erythrocytes were precipitated from goat blood and lysed by 10 volumes of water. Then, 0.1 ml clarified erythrocytes were inoculated i.v. into embryonated eggs. The eggs were incubated at 33.5°C for 7 days. Embryos that died between 2 and 7 dpi were dissociated in 1 ml of PBS by syringe and clarified by centrifugation at 1,000 *g*. Then, 0.1 ml of the supernatant was inoculated into a T25 flask with C6/36 cells and 5 ml 2% FBS^+^ (Gibco) MEM (Gibco). The C6/36 cells were cultured at 28°C for 7 days. Then, 1 ml of the supernatant from C6/36 culture was inoculated into a T25 flask of BHK‐21 with 5 ml 2% FBS^+^ MEM. If CPE was observed, the flask with BHK‐21 was stored at 4°C until further analysis. If no CPE was observed, the culture was blind passaged up to three times before being called negative.

### 2.4 Complete sequencing of the BTV genome

#### 2.4.1 Viral genomic dsRNA extraction

BTV (YN/2017)‐infected BHK‐21 cells (48 hpi) were collected by scraping and centrifugation. The cell pellet was suspended in 100 μl medium and lysed by freezing and thawing twice, followed by incubation with RNase A (Takara) at 37°C for 12 hr to delete cellular ssRNA (Yang et al., 2012). Then, the viral dsRNA was extracted by TRIzol (Thermo), precipitated with isopropanol and washed with 75% ethanol according to the manufacturer's protocol. The dsRNA was collected by centrifugation (4°C, 5200 g, 5 min) and air‐dried.

#### 2.4.2 Viral genomic cDNA synthesis

Complete viral genomic RNA was amplified by FLAC methods. Both the procedure and the primers used were in accordance with methods by Maan (Maan et al., 2011). Briefly, the 3’ end of the viral dsRNA was covalently linked with anchor primer by T4 RNA ligase 1 (NEB) overnight. The anchored dsRNA was purified by an RNA extraction kit (Takara) and used to synthesize cDNA with Superscript III (Thermo).

#### 2.4.3 Amplification and sequencing of the complete virus genome

Viral genomic cDNA was denatured at 95°C and renatured to dsDNA through gradually cooling from 95°C to 25°C. Ten microlitres of dsDNA and 4 μl of 10 μM primer 5‐15‐1 (Maan et al., 2007) were added to a 100 μl PrimeSTAR‐GXL (Takara, Japan) PCR system. Amplification was carried out after denaturation at 95°C for 2 min, followed by 40 cycles of denaturation for 10 s at 95°C, annealing for 10 s at 55°C, extension for 4 min at 68°C and a final extension for 1 min at 68°C.

Amplified complete genomic DNA was sent to the MAGIGEN Company for high‐throughput sequencing by a HiSeq 2000 system (Illumina) and *SOAPdenovo* software.

## 3 RESULTS

### 3.1 Serologic investigation of yaks in Shangri‐La

A total of 507 yak serum samples were collected by the local ADCC during 2014 to 2017 in five villages within the Shangri‐La district. As shown in Table 1, the C‐ELISA tests suggested that a few yaks from three villages had been infected by BTV during 2014 to 2016, and no BTV infection was discovered in the other two villages (Dongwang and Xiaozhongdian). In other words, there were random and sporadic BTV infections among yaks in Shangri‐La during 2014 to 2016.

**Table 1:** Serological investigation of yaks in Shangri‐La during 2014–2017

Village or small town	Geographic coordinate	Altitude	Seroprevalence of BTV (*n* tested)			
			2014	2015	2016	2017
Dongwang	28.58 N, 99.69 E	2,889	0 (24)	0 (25)	0 (25)	0 (25)
Geza	28.06 N, 99.77 E	3,227	10% (30)	0 (30)	20% (30)	0 (28)
Jiantang	27.80 N, 99.71 E	3,290	0 (25)	0 (24)	22% (23)	0 (24)
Luoji	27.80 N, 100.20 E	2,183	22% (27)	0 (25)	0 (25)	0 (24)
Xiaozhongdian	27.59 N, 99.79 E	3,220	0 (22)	0 (25)	0 (23)	0 (23)

### 3.2 Serologic investigation of goats in a nearby river valley

Three goat farms in one of the river valleys in Nixi (approximately 28.05 N, 99.51 E) in Shangri‐La were selected for serologic investigation. The three farms were located on a hilltop, a hillside and the foot of the hill. Goats were investigated because they are popular ruminants raised in this rocky area. The serologic investigation by C‐ELISA suggested that there were notably high BTV infection rates (35.48%–65.63%) among the flocks of goats in the river valley (Table 2). The clearly different BTV infection rates between the yaks and goats reported here might be attributed to the difference in environmental temperature because a relatively moist and warm climate promotes the propagation of *Culicoides*, which is a critical factor in BTV transmission among ruminants.

**Table 2:** Serological investigations at three goat farms in a valley in Shangri‐La in 2017

Farms	Altitude	Number of goats		Positive ratio of anti‐BTV sera (%)
		Total	Positive	
1	2,800	31	11	35.48
2	2,400	32	21	65.63
3	2,000	30	15	50.00

### 3.3 Serologic surveillance and virus isolation

Two surveillance sites were established in the other two river valleys in Shangri‐La. Site 1 and site 2 were located at 27.10 N/100.12 E and 28.17 N/99.70 E, respectively and had altitudes of 2,000 m and 2,800 m, respectively. The linear distance between the two sites was approximately 126 km.

A total of 20 goats identified as BTV negative by C‐ELISA were chosen as sentinel animals and kept at the two sites. The sentinel animals were continuously monitored, and the serum and EDTA^+^ blood samples were collected every 10–14 days between May and September of 2017. All the sera collected were tested by C‐ELISA, and the results (Table 3) showed that 5 sentinel animals were identified as BTV‐infected animals (PI ≥ 50%) and the others were dubious (0 < PI <50%). Since they were clearly positive, the samples from animals #4 and #5 were not collected after the 24 July. Meanwhile, all the EDTA^+^ blood samples collected was used to isolate virus through the ECB virus isolation system. As a result, 13 BTV isolates were isolated from 10 sentinel animals (Table 3).

**Table 3:** Serological surveillances of sentinel goats at two sites from 28 May to 18 September 2017

Site	No. of animal	Date (day/month)								
		28/5 (%)	14/6 (%)	27/6 (%)	10/7 (%)	24/7 (%)	7/8 (%)	21/8 (%)	4/9 (%)	18/9 (%)
1	1	10.08	0.00	36.41	3.54	16.61	17.32	7.83	0.83	17.44
	2	11.39	5.45	11.26	13.76	21.29	25.71	9.44	14.53	20.04
	3	6.32	8.83	1.02	6.48	16.75	13.41	18.07	9.03	26.05
	4	**17.62**	88.20	91.25	83.71	–	–	–	–	–
	5	**13.76**	55.09	84.34	87.76	–	–	–	–	–
	6	11.69	3.55	5.45	25.48	35.70	24.80	25.03	21.45	32.55
	7	11.90	42.30	28.88	29.40	**28.72**	**62.36**	50.73	61.48	57.27
	8	10.33	18.36	**12.02**	29.69	30.43	30.19	21.56	22.12	28.22
	9	1.39	57.43	**57.96**	59.91	60.21	**63.32**	60.60	68.76	67.28
	10	1.64	3.96	15.23	27.38	34.21	22.39	15.04	14.92	19.64
2	11	6.90	7.38	20.15	0.00	24.67	3.54	15.34	16.43	20.87
	12	3.46	10.60	18.53	26.78	**25.07**	24.49	17.13	27.92	22.34
	13	5.91	20.04	**18.45**	17.40	22.79	24.34	16.88	27.17	22.01
	14	9.03	29.53	17.19	17.24	26.16	20.13	32.73	27.70	24.68
	15	11.99	10.60	2.57	22.56	34.60	29.56	**15.38**	**38.75**	22.64
	16	3.33	6.01	28.10	17.32	22.87	26.30	**25.17**	18.24	27.23
	17	10.62	6.37	22.37	37.54	45.10	38.94	28.43	35.79	17.32
	18	2.84	0.00	5.42	**14.20**	20.41	28.00	88.14	90.73	90.89
	19	10.37	12.50	23.32	19.67	29.90	23.58	14.60	24.33	28.83
	20	14.54	12.43	18.45	31.41	42.44	31.54	16.24	23.41	23.75

Note

Results are presented as the percent inhibition (PI) as determined by C‐ELISA. Animals No. 4 and No. 5 ceased to be surveyed because their sera were obviously positive. Virus was isolated from synchronously collected EDTA blood samples, as shown in bold.

The isolated virus was used for SNT of sera from the five positive sentinel goats, and the titres were 64, 64, 45, 32 and 128. The high level of titres confirmed that our BTV isolates reliably came from the sentinel goats.

### 3.4 A strain of BTV‐21 was identified

To identify the BTV strains, primary sequencing was performed. A Seg1 fragment from 400 to 828 bp was amplified by one‐step RT‐PCR kit with the universal primers Bs1p3f and Bs1p3r, and the 184 bp middle sequence (524–707 bp) exhibited characteristics of BTV serotype 21.

EDTA^+^ blood samples from 31 yaks and 10 sheep were collected from the highland area near the two surveillance sites in 2017 and tested by RT‐PCR. BTV nucleic acids were detected in the blood of 10 yaks and 2 sheep. For Seg1 fragment alignment, the samples from the positive yaks, positive sheep, 20 sentinel goats and four isolates from Shangri‐La and representative strains (Table S2) isolated previously in China were used. The alignment of the 184 bp sequences of Seg1 showed that our isolates were identical (99%–100%) with the BTV strains from some goats, yaks and sheep in Shangri‐La but showed obvious variation from the former BTV strains isolated in China (Figure 1).

### 3.5 The isolated BTV was homologous with BTV‐21 in Indonesia

Since all the isolates showed accordant properties by SNT and Seg1 fragment sequencing, one of the isolates (Shangri‐La/2017/763), also named BTV stain YN/2017, was sequenced completely. The Seg1 to Seg10 sequences of YN/2017 were registered in GenBank with accession numbers from MK250956 to MK250965.

**Figure 2:**
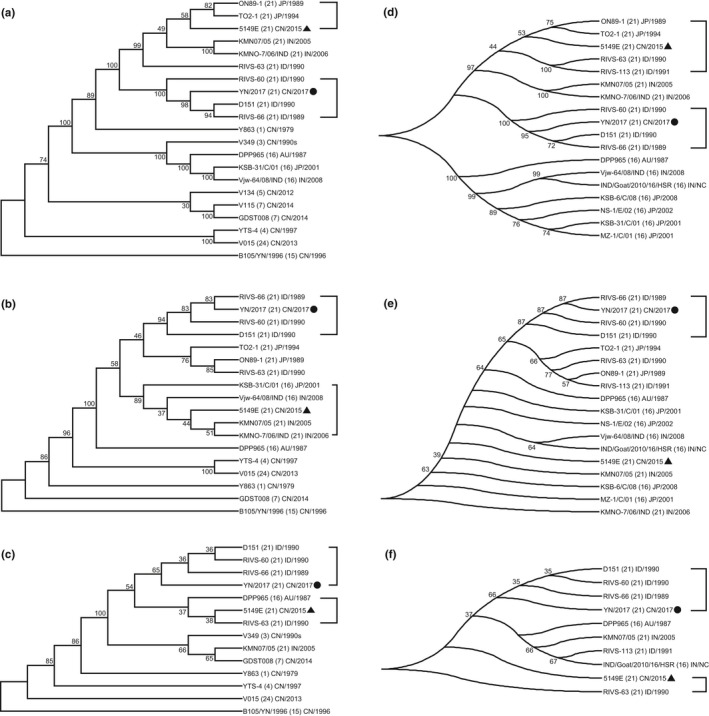
Phylogenetic analysis of the relationship between YN/2017 and other BTV strains. The homologies between YN/2017 and the domestic BTV strains were analysed by the amino acid sequences of VP2 (a), VP5 (b) and VP7 (c), respectively. The homologies between YN/2017 and the represents of related strains worldwide were analysed by the amino acid sequences of VP2 (d), VP5 (e) and VP7 (f), respectively. All the sequence alignments were performed by *MEGA‐X*, and the evolutionary trees were built by the neighbour‐joining method (bootstrap = 1,000). The information for strain, serotype, country and year collected is labelled

Online BLAST analysis (NCBI) for the complete sequences of 10 segments from YN/2017 showed that this BTV strain belongs to serotype 21 and is almost identical to three BTV strains (D151, RIVS‐66, RIVS‐60) isolated in West Java in Indonesia in 1989–1990 (Table 4). Overall alignment was performed among domestic BTV strains, including the first completely sequenced BTV‐21 (5149E) strain in China, and several related overseas strains from Indonesia, India and Australia. As a result, our strain (YN/2017) was the most homologous to three Indonesian BTV‐21 strains and had apparent differences from other domestic BTV strains sequenced previously (Figure 2, a–c). It has also been suggested that the newly isolated BTV‐21 strain YN/2017 (Shangri‐La, Yunnan Province, 2017) has no direct relation to strain 5149E (Long‐an, Guangxi Province, 2015) but that they are both homologous with BTV strains from Indonesia (Figure 2). The former is more homologous with strains D151, RIVS‐66 and RIVS‐60, and the latter is more homologous with Japanese strains ON89‐1 and TO2‐1 as well as Indonesia strains RIVS‐63 and RIVS‐113 (Figure 2, d and f).

**Table 4:** Characteristics of the complete genome segments and their deduced proteins from BTV‐21 strain YN/2017

Segment (size: bp)	Range of ORFs	Protein (size: aa)	GenBank accession number	Closest strain (serotype, country)	Sequence identity of CDS (%)
1 (3,944)	12:3,920	VP1 (1,302)	MK250956	D151 (21, INA)	99.9
2 (2,922)	18:2,885	VP2 (955)	MK250957	D151, RIVS‐66 (21, INA)	99.9
3 (2,772)	18:2,723	VP3 (901)	MK250958	RIVS‐66, RIVS‐60 (21, INA)	100
4 (1,981)	9:1,943	VP4 (644)	MK250959	RIVS‐66 (21, INA)	99.5
5 (1,768)	35:1,693	NS1 (552)	MK250960	D151, RIVS‐66 (21, INA)	99.9
6 (1,637)	29:1,609	VP5 (526)	MK250961	D151, etc^a^ (21, INA)	100
7 (1,156)	18:1,067	VP7 (349)	MK250962	D151, etc^a^ (21, INA)	100
8 (1,125)	20:1,084	NS2 (354)	MK250963	D151, RIVS‐66 (21, INA)	99.9
9 (1,052)	16:1,008	VP6 (330)	MK250964	RIVS‐66, RIVS‐60 (21, INA)	99.8
	185:418	NS4 (77)		D151, etc^a^ (21, INA)	100
10 (822)	20:709	NS3 (229)	MK250965	D151, RIVS‐60 (21, INA)	100
	61:709	NS3a (216)		D151, etc^a^ (21, INA)	100

a There are three strains D151, RIVS‐66 and RIVS‐60.

Furthermore, 10 isolates from 10 sentinel goats were analysed. A group of Seg2 fragments with 999 bp were amplified by a Pfu PCR system with primers S2‐F and S2‐R. Eight of the PCR products with clear, bright target bands were sent for Sanger sequencing using primers S2‐F and S2‐R, while the other two PCR products with dim target bands were not sequenced. A multiple sequence alignment was performed for the eight confirmed DNA sequences with 867 bp (1654–2520 bp) and the corresponding Seg2 sequences of BTV strains YN/2017 and 5149E. The results showed that all 8 isolates had 100% identity with YN/2017 but showed apparent differences with 5149E (data not shown).

## 4 DISCUSSION

Thus far, a total of 14 serotypes of BTV (1, 2, 3, 4, 5, 7, 9, 11, 12, 15, 16, 21, 23 and 24) have been isolated in tropical and subtropical areas of China, and all serotypes except type 7 have been found in Yunnan Province (Kirkland et al., 2002; Yang et al., 2017). Recently, BTV traces were found on the Qinghai‐Tibetan Plateau in Northwest China, where the average altitude is more than 4,000 metres and the average temperature is approximately 0°C. Additionally, a 13.3% seroprevalence of anti‐BTV IgG in yaks in Tibet (Ma et al., 2017) and one case of BTV infection in a yak in Qinghai Province (Hu et al., 2016; Li et al., 2015) have been reported. However, no RNA sequence information for these BTV strains has been reported. Hence, it is meaningful to investigate yaks and BTV in Shangri‐La, Yunnan Province.

The Shangri‐La plateau, which is located in Yunnan Province in Southwest China, is located in the subtropics and adjacent to the Qinghai‐Tibetan Plateau and has an average altitude of 3,450 m. Similar to the Alps, the Shangri‐La plateau is rugged and has abundant water resources. Several rivers, including the Jinsha River, which is upstream of the Changjiang River, flow across this plateau.

This study was the first that we are aware of to identify BTV infection in yaks and endemic BTV infection in the relatively warm river valleys in Shangri‐La. Serologic investigations show that there was a 0%–22% seroprevalence of anti‐BTV IgG in yaks in five villages on the highland during 2014 to 2017 (Table 1), and there was a 50%–65.63% seroprevalence at three goat farms in the nearby valley area (Table 2). However, the direct viral origin of BTV in local yaks is unknown. It is possible that the BTV was transmitted from the viral reservoirs in the valleys to the yaks at higher altitude by *Culicoides* vectors because the distance between yak farms in the mountain areas and goat farms in the valleys was less than 20 kilometres. In addition, some *Culicoides* have been collected in the valleys, and the dominant species have been identified as *C. nielamensis*,* C. tainanus* and *C. obsoletus*, but the BTV vectors were not confirmed in this study. Certainly, the possibility that the BTV comes from the Qinghai‐Tibetan Plateau by traded ruminants cannot be excluded, although traffic would be very difficult.

Our preliminary investigation suggested that BTV existed in Shangri‐La. Subsequently, 20 sentinel goats were placed at two sites in two valleys in 2017, and several BTV isolates were isolated from the sentinel goats. All isolates were identified as the same strain, and one of the isolates (named YN/2017) was sequenced completely. Interestingly, the genome of BTV strain YN/2017 was almost identical to those of three Indonesia BTV‐21 strains (D151, RIVS‐66 and RIVS‐60; Table 4) isolated in West Java during 1989 to 1990 (Firth et al., 2017). However, it was clearly different from the domestic strain 5149E, which was isolated in southern China in 2015 and was the first completely sequenced BTV‐21 strain in China (Qin et al., 2018). There are 51 differences between the VP2 amino acid sequences of YN/2017 and 5149E. Similar to the Indian strain KMNO‐7 (Shafiq et al., 2013), 5149E has the Seg2 RNA of serotype 21 and the Seg6 RNA of serotype 16, but it is more homogenous with Japanese strains (Qin et al., 2018).

**Figure 3:**
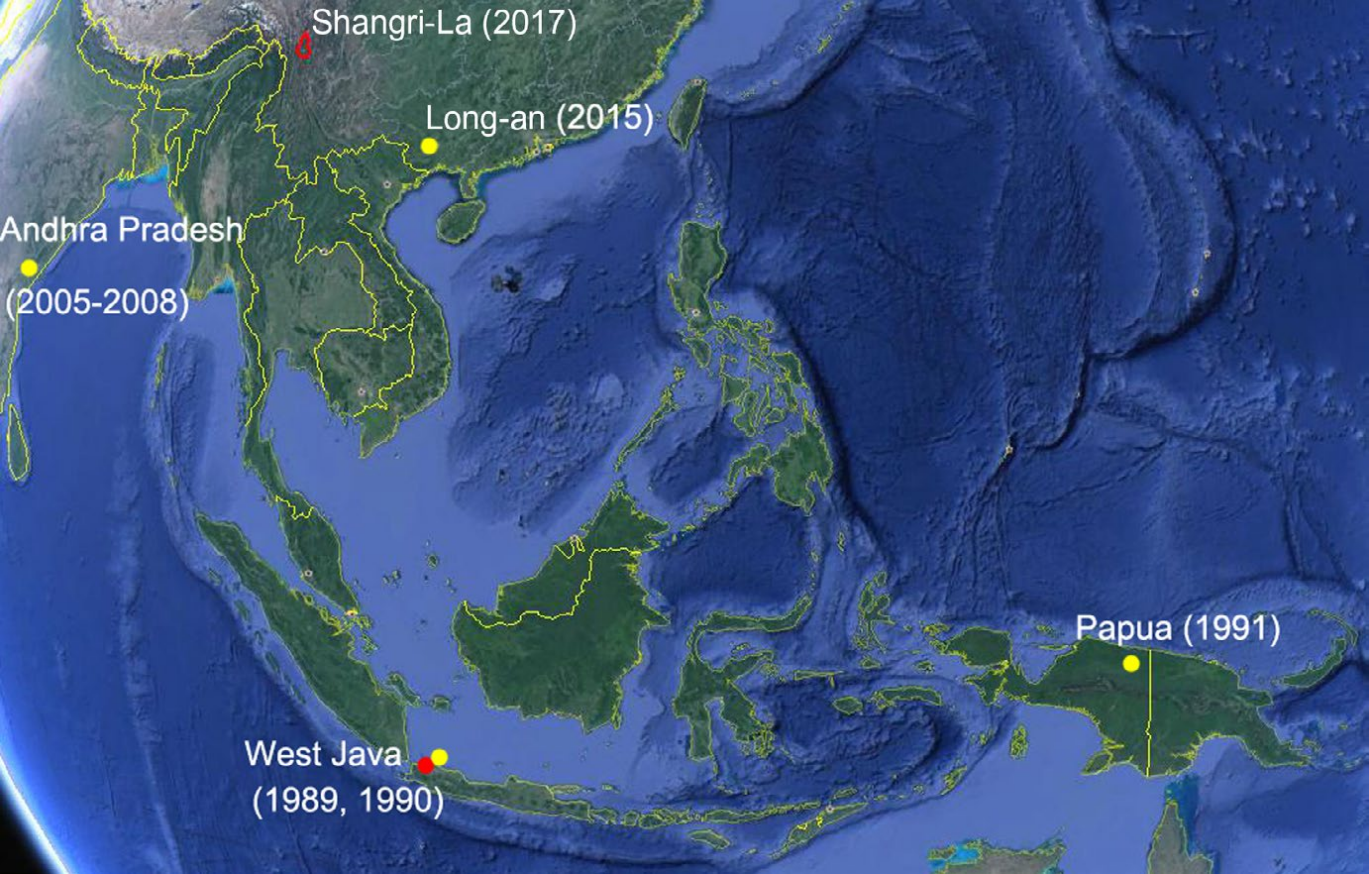
Sites of collection of the BTV‐21 YN/2017 and 5149E strains as well as their homogenous strains. The sites of collection of YN/2017 and its homogenous strains were labelled with red dots, while the sites of collection of 5149E and its homogenous strains were labelled with yellow dots. The map was drawn based on a Google map

It is clear that strain YN/2017 and the Indonesian strains have direct consanguinity. However, there is no direct proof that would explain the transmission direction of this strain between China and Indonesia. However, considering the international trade of animals, it is possible that the BTV‐21 strain YN/2017 came from Southeast Asia somehow (Figure 3). As we know, China does not export cattle and sheep out of the country, except that Inner Mongolia in northern China may export sheep to the Middle East. In contrast, Australia has a high rate of exportation of cattle and sheep. It largely exports cattle to Indonesia and Vietnam and has recently begun exporting cattle and sheep to China. Since Australia does not directly export animals to Yunnan in China and the animals are monitored by Chinese customs and quickly slaughtered, it is unlikely that the BTV strain YN/2017 came from Australia directly. On the other hand, Myanmar borders Yunnan, China and exports cattle to Yunnan directly, and Vietnam is bordered by both Yunnan and Guangxi and directly exports cattle to these provinces as well. Therefore, the BTV‐21 strain YN/2017 in Shangri‐La may have come from Indonesia or Australia through the countries of Myanmar, Vietnam or Laos (Figure 3).

Further studies are needed to answer the remaining questions, including the relationship between BTV strains from Shangri‐La and the Qinghai‐Tibetan Plateau, and the reasons why this strain has maintained a very low incidence of mutation over nearly three decades.

## ETHICS STATEMENT

The processes of blood collections from animals (yaks, goats and sheep) were approved by their hosts, and the animal health was protected. The study was approved by the Ethics Committee of Yunnan Animal Science and Veterinary Institute.

## References

[tbed13292-bib-0001] Clavijo, A. , Heckert, R. A. , Dulac, G. C. , & Afshar, A. (2000). Isolation and identification of bluetongue virus. Journal of Virological Methods, 87, 13–23. 10.1016/S0166-0934(00)00150-6 10856748

[tbed13292-bib-0002] Firth, C. , Blasdell, K. R. , Amos‐Ritchie, R. , Sendow, I. , Agnihotri, K. , Boyle, D. B. , … Walker, P. J. (2017). Genomic analysis of bluetongue virus episystems in Australia and Indonesia. Veterinary Research, 48, 82 10.1186/s13567-017-0488-4 29169390 PMC5701493

[tbed13292-bib-0003] Gibbs, E. P. , & Greiner, E. C. (1994). The epidemiology of bluetongue. Comparative Immunology, Microbiology and Infectious Diseases, 17, 207–220. 10.1016/0147-9571(94)90044-2 8001346

[tbed13292-bib-0004] Hu, G. W. , Shen, Y. L. , Zhao, Q. B. , Chao, Y. L. , Kan, W. , Zhou, J. Z. , … Cai, J. S. (2016). Preliminary investigation on abortion of yaks in part of Qinghai Province. Chinese Journal of Veterinary Medicine, 52, 3–5.

[tbed13292-bib-0005] Jenckel, M. , Breard, E. , Schulz, C. , Sailleau, C. , Viarouge, C. , Hoffmann, B. , … Zientara, S. (2015) Complete coding genome sequence of putative novel bluetongue virus serotype 27. Genome Announcements, 3(2), e00016–15 25767218 10.1128/genomeA.00016-15 PMC4357740

[tbed13292-bib-0006] Kirkland, P. D. , Zhang, N. , Hawkes, R. A. , Li, Z. , Zhang, F. , Davis, R. J. , … Hunt, N. T. (2002). Studies on the epidemiology of bluetongue virus in China. Epidemiology and Infection, 128, 257–263. 10.1017/S0950268801006525 12002544 PMC2869819

[tbed13292-bib-0007] Li, J. , Li, K. , Shahzad, M. , Han, Z. , Nabi, F. , Gao, J. , & Han, J. (2015). Seroprevalence of Bluetongue virus in domestic yaks (Bos grunniens) in Tibetan regions of China based on circulating antibodies. Tropical Animal Health and Production, 47, 1221–1223. 10.1007/s11250-015-0853-0 26017752

[tbed13292-bib-0008] Ma, J. G. , Zhang, X. X. , Zheng, W. B. , Xu, Y. T. , Zhu, X. Q. , Hu, G. X. , & Zhou, D. H. (2017). Seroprevalence and Risk Factors of Bluetongue Virus Infection in Tibetan Sheep and Yaks in Tibetan Plateau, China. BioMed Research International, 2017, 1–5 10.1155/2017/5139703 PMC5420416 28512638

[tbed13292-bib-0009] Maan, S. , Maan, N. S. , Nomikou, K. , Batten, C. , Antony, F. , Belaganahalli, M. N. , … Mertens, P. P. (2011). Novel bluetongue virus serotype from Kuwait. Emerging Infectious Diseases, 17, 886–889. 10.3201/eid1705.101742 21529403 PMC3321788

[tbed13292-bib-0010] Maan, S. , Rao, S. , Maan, N. S. , Anthony, S. J. , Attoui, H. , Samuel, A. R. , & Mertens, P. P. (2007). Rapid cDNA synthesis and sequencing techniques for the genetic study of bluetongue and other dsRNA viruses. Journal of Virological Methods, 143, 132–139. 10.1016/j.jviromet.2007.02.016 17433453

[tbed13292-bib-0011] Maclachlan, N. J. (2011). Bluetongue: History, global epidemiology, and pathogenesis. Preventive Veterinary Medicine, 102, 107–111. 10.1016/j.prevetmed.2011.04.005 21570141

[tbed13292-bib-0012] Qin, S. , Yang, H. , Zhang, Y. , Li, Z. , Lin, J. , Gao, L. , … Wu, J. (2018). Full genome sequence of the first bluetongue virus serotype 21 (BTV‐21) isolated from China: Evidence for genetic reassortment between BTV‐21 and bluetongue virus serotype 16 (BTV‐16). Archives of Virology, 163, 1379–1382. 10.1007/s00705-018-3718-9 29392498

[tbed13292-bib-0013] Reed, L. J. , & Muench, H. (1938). A simple method of estimating fifty percent endpoints. American Journal of Epidemiology, 27, 493–497.

[tbed13292-bib-0014] Roy, P. (2007) Orbiviruses In Fields B. N., Knipe D. M., & Howley P. M. (eds), Fields Virol. 5th edn 54, (pp. 2541–2568). Philadelphia, PA: Wolters Kluwer Health/Lippincott Williams & Wilkins.

[tbed13292-bib-0015] Shafiq, M. , Minakshi, P. , Bhateja, A. , Ranjan, K. , & Prasad, G. (2013). Evidence of genetic reassortment between Indian isolate of bluetongue virus serotype 21 (BTV‐21) and bluetongue virus serotype 16 (BTV‐16). Virus Research, 173, 336–343. 10.1016/j.virusres.2013.01.009 23353779

[tbed13292-bib-0016] Xiao, L. , Wang, M. L. , Niu, M. C. , Wang, J. P. , Gao, L. , Li, H. C. , & Zhu, J. B. (2011). The research of anti‐bluetongue virus serum of yaks in Shangri‐La, Songpan and Ruoergai. Shanghai Xumu Shouyi Tongxun, 56, 26–27.

[tbed13292-bib-0017] Yang, H. , Xiao, L. , Wang, J. , Meng, J. , Lv, M. , Liao, D. , … Li, H. (2017). Phylogenetic Characterization Genome Segment 2 of Bluetongue Virus Strains Belonging to Serotypes 5, 7 and 24 Isolated for the First Time in China During 2012 to 2014. Transboundary and Emerging Diseases, 64, 1317–1321. 26865326 10.1111/tbed.12479

[tbed13292-bib-0018] Yang, H. , Zhu, J. , Li, H. , Xiao, L. , Wang, J. , Li, N. , … Kirkland, P. D. (2012). Full genome sequence of bluetongue virus serotype 4 from China. Journal of Virology, 86, 13122–13123. 10.1128/JVI.02393-12 23118453 PMC3497667

