# Supplementary material for: The serologic investigation and viral isolation of bluetongue virus in Shangri‐La in Southwest China
Source: Transbound Emerg Dis. 2019 Jul 22;66(6):2353–61. doi: 10.1111/tbed.13292 (PMC6899809; doi:10.1111/tbed.13292)
Supplement: Supplementary file 1 [file TBED-66-2353-s001.doc]

**SUPPLEMENTARY TABLE 1** The animals used in this study

| Research order | Animals | Samples collected for research | | | Experiments | Related Sections |
| --- | --- | --- | --- | --- | --- | --- |
|  |  | Specimens | Sites | Times |  |  |
| 1 | Yaks | Serum | 5 villages | 2014-2017 | C-ELISA | 3.1 |
| 2 | Goats | Serum | 3 farms in Nixi | 2017 | C-ELISA | 3.2 |
| 3 | Sentinel goats | Serum, Blood | 2 sites in Nixi | 2017 | C-ELISA, Viral isolation, SNT | 3.3, 3.4 |
| 4 | Yaks/Sheep | Blood | Xiaozhongdian, Nixi | 2017 | Sequencing and alignment for BTV Seg1 fragments | 3.4 |
