# Supplementary material for: The serologic investigation and viral isolation of bluetongue virus in Shangri‐La in Southwest China
Source: Transbound Emerg Dis. 2019 Jul 22;66(6):2353–61. doi: 10.1111/tbed.13292 (PMC6899809; doi:10.1111/tbed.13292)
Supplement: Supplementary file 2 [file TBED-66-2353-s002.doc]

**SUPPLEMENTARY TABLE 2** The background of some BTV strains isolated before in China

| Strain/Isolate | Serotype | Origin | | | Registed information  in GenBank |
| --- | --- | --- | --- | --- | --- |
|  |  | Animal | Location | Year |
| V005 | 3 | Cattle | Shizong/YN | 2012 | None |
| V007 | 12 | Cattle | Nanning/GX | 2014 | None |
| V008 | 9 | Cattle | Dehong/YN | 2013 | None |
| V015 | 24 | Cattle | Dehong/YN | 2013 | Seg2, Seg3, Seg6, Seg7 |
| V016 | 16 | Cattle | Dehong/YN | 2013 | None |
| V028 | 21 | Cattle | Puer/YN | 2013 | None |
| V033 | 21 | Cattle | Dehong/YN | 2013 | None |
| V037 | 2 | Cattle | Puer/YN | 2013 | None |
| V061 | 15 | Cattle | Puer/YN | 2013 | None |
| V089 | 7 | Cattle | ---/GD | 2014 | Seg2 |
| V134 | 5 | Cattle | Shizong/YN | 2012 | Seg2 |
| V1863 | 1 | Sheep | Shizong/YN | 1979 | None |
| dh/2018 | 1 | Cattle | Dehong/YN | 2018 | None |

Note: the locations were shown as county/province, and the provinces were Yunnan Province (YN), Guangdong Province (GD), and Guangxi Province (GX).
